# Serum Oxytocin Level Correlates With Gut Microbiome Dysbiosis in Children With Autism Spectrum Disorder

**DOI:** 10.3389/fnins.2021.721884

**Published:** 2021-10-01

**Authors:** Minshi Huang, Kevin Liu, Zhen Wei, Zhe Feng, Jierong Chen, Jie Yang, Qin Zhong, Guobin Wan, Xue-Jun Kong

**Affiliations:** ^1^Department of Child Psychology and Rehabilitation, Affiliated Shenzhen Maternity and Child Healthcare Hospital, Southern Medical University, Shenzhen, China; ^2^Athinoula A. Martinos Center for Biomedical Imaging, Massachusetts General Hospital and Harvard Medical School, Charlestown, MA, United States; ^3^Department of Medicine and Psychiatry, Beth Israel Deaconess Medical Center and Harvard Medical School, Boston, MA, United States

**Keywords:** autism spectrum disorder, oxytocin, gut microbiota, sex-dependence, dysbiosis

## Abstract

To investigate the levels of serum oxytocin (OT) in children with autism spectrum disorder (ASD) and explore the association between OT levels and gut microbiota relative abundances, we recruited 39 children with ASD children–mother dyads and 44 healthy controls. Serum OT levels were determined *via* enzyme-linked immunosorbent assay and gut microbiota abundances were determined by 16S rRNA sequencing. We found that the OT level of ASD was lower than the healthy control group overall (*P* < 0.05). Furthermore, we present preliminary evidence of gut microbiome dysbiosis observed among children with ASD to lower levels of OT based on correlational analysis between serum OT and specific gut microbiota abundances (*P* < 0.05). We also found sex-related differences in serum OT levels and GIS index (*P* < 0.05). However, the generalizability of findings relevant to females with ASD require further validation in future studies involving larger sample sizes and balanced sex distributions due to the small number of females involved in this study. Nonetheless, these new findings further our understanding of the effects of low serum OT levels among individuals with ASD, which provides preliminary evidence in hopes of guiding future study design or mechanistic studies. The findings of the present study may be suggestive of potential ASD subtypes based on ASD severity and gut microbiome composition that may facilitate the prediction of the therapeutic responses of OT among those with ASD.

## Introduction

Autism spectrum disorder (ASD) is a neurodevelopmental disorder with two core symptoms: social communication deficits and restricted, repetitive patterns of behavior (American Psychiatric Association, 2013; Lai et al., 2014). The prevalence rate reported by CDC in 2020 was 18.5 per 1000 individuals (one in 54) (Baio et al., 2018). Furthermore, ASD has become a serious public health issue due to its apparent rise in prevalence and poor prognosis (Lavelle et al., 2014). Yet, the pathogenesis of ASD remains largely unclear and there is currently no known effective treatment.

Oxytocin (OT) is a multi-function neuropeptide which has attracted significant attention in recent years due to its neuro-modulatory effects. In the early developmental stage, OT plays a role in the modulation of GABA functional status *via* chloride transporters to maintain excitation–inhibition balance. Its nasal administration has shown some initial promising efficacy in improving the core symptoms of ASD (Ooi et al., 2016; DeMayo et al., 2017; Peñagarikano, 2017; Montag et al., 2020). OT is a highly conserved peptide of nine amino acids synthesized by nerve cells in the paraventricular nucleus and the supraoptic nucleus of the hypothalamus and is released into the blood *via* the axon endings in the posterior lobe of the pituitary gland. In addition to being released into the circulatory system, OT receptors have a wide distribution in the brain (Gulliver et al., 2018). OT plays an important role in a variety of physiological phenomena, such as regulating digestion, blood pressure, heart rate, and pain (Lee et al., 2009). Evidence from animal studies and several human clinical trials suggest that OT treatment may improve learning, emotional, and social behaviors, such as increasing in-group trust, eye contact, emotion recognition, and social cognition (Modahl et al., 1998; Ooi et al., 2016; DeMayo et al., 2017; Peñagarikano, 2017; Montag et al., 2020). However, the efficacy and therapeutic response of different clinical trials showed variable results. We hypothesize that beneficial responses to OT treatment are only apparent for a subset of ASD individuals. A meta-analysis of 12 randomized controlled trials (RCTs) of OT on social recognition of ASD patients showed no evidence of significant efficacy of OT compared to placebo; however, the consistency of such overall evidence was low due to substantial inter-trial heterogeneity, including variations in study design, patient characteristics, primary outcome assessments, OT dosage, and duration of OT administration. In addition, individuals with ASD may vary in the functionality of their endogenous OT system and it remains likely that clinically significant responses can only be observed within particular ASD subgroups that are more genetically prone to dysfunctions in OT signaling (Ooi et al., 2016). Oxytocin is highly correlated with gut microbiome *via* immune–endocrine-brain signaling networks (Erdman and Poutahidis, 2016). The treatment with probiotics *L. reuteri* selectively rescues social deficits *via* induction of endogenous OT (Sgritta et al., 2018). Thus, it is important to study the associations between OT level and the abundances of specific gut microbiota in addition to clinical parameters.

Previous studies found that blood OT level is highly correlated with brain OT levels despite inconsistent findings of relative OT levels between children with ASD and healthy children (Modahl et al., 1998; Cochran et al., 2013; Parker et al., 2014; Aita et al., 2019). Such inconsistencies likely arise from reported differences in age, sex, severity, and cognitive function level among subject populations. Therefore, further investigation of such findings between blood and brain OT levels in ASD is warranted. Moreover, correlational findings based on therapeutic response to administration of nasal OT treatment have not been studied. This study aims to compare serum OT levels in children with ASD, their mothers, and a cohort of healthy controls to further identify associations between serum OT levels, ASD severity, and gut microbiota abundances. We also assessed for potential factors that may influence serum OT levels in children with ASD to further understand the role of OT in ASD.

## Materials and Methods

### Ethics Statement

This study was approved by the Ethics Committee of Shenzhen Maternity and Child Healthcare Hospital. The parents or legal guardians of the participants were informed of the purposes and detailed procedures of the study and gave informed consent before initiation of the experimental procedures.

### Participants

Thirty-nine children with ASD (32 male and 7 females) between the ages of 3 and 7 years were enrolled from Shenzhen Maternity and Child Healthcare Hospital between March and June 2018. ASD children were diagnosed by an experienced child psychiatrist according to the Diagnostic and Statistical Manual of Mental Disorders-fifth edition criteria (DSM-5). All ASD participants were first seen by the psychiatrist, and then their behavioral assessments were performed by two practiced psychometrists. In addition, 38 mothers whose children were included in the ASD group were recruited as the mother control group.

Forty-four children with normal development were enrolled voluntarily in a kindergarten and underwent physical examination in our hospital in May 2018.

The exclusion criteria for all subjects include:

• Suffering from genetic, metabolic, and/or endocrine diseases that are not known to be associated with ASD;
• Infection, inflammation, allergy, or other clinical manifestations in the past week;
• Taking probiotics in the past week;
• Currently receiving other medical treatment;
• Receiving OT treatment for more than 3 months.

### 6-Item Gastrointestinal Severity Index

The 6-Item Gastrointestinal Severity Index (6-GSI) is a modified version of gastrointestinal (GI) Severity Index, which is used to assess gastrointestinal symptoms, including only the first six items (constipation, diarrhea, stool consistency, stool smell, flatulence, and abdominal pain). The higher the score, the more severe the symptoms (Schneider et al., 2006).

### Serum Oxytocin Measurement

Blood samples were collected between 9:00 and 11:00 a.m. after the participants had rested for at least 30 min. Three to five milliliters of venous whole blood was drawn into a BD Vacutainer^®^ containing the inert colloidal coagulant, then inverted and mixed for 5–8 times immediately after collection, allowing samples to clot for 30 min before centrifugation. Samples were centrifuged for 10 min at approximately 3000 × *g*. Serum was then collected and stored at −80°C. The OT concentrations were measured using commercially available Human Oxytocin ELISA kits (CD-101605-ELISA, from Chundu Bio, Wuhan, China^1^). Standards or samples were first added to the appropriate microtiter plate wells and horseradish peroxidase (HRP)-conjugate reagent was then added to each well and incubated. Each well is then aspirated and washed for a total of five repetitions. Substrate solutions are then added to each well and the color of each well is expected to change from blue to yellow. The optical density (OD) at 450 nm is read using a microtiter plate reader (BEP III analysis system, Siemens Healthcare Diagnostics Products GmbH, Germany) within 15 min. The standard curve is plotted and the amount of OT in each unknown sample is interpolated. The sensitivity of this assay is 1.0 pg/ml and the coefficient of variation within and between plates is less than 15%.

### Intestinal Microbiome Sampling

#### Stool Sample Collection

Stool samples were collected by an informed parent or caregiver of the study participant in accordance with the protocol provided along with the stool collection kit (Precidiag Inc.). Collected samples were stored at room temperature for a maximum of 2 days and in a −80°C freezer upon reception at the research facility prior to transfer to Beijing Boao Medical Laboratory for sample processing.

#### DNA Extraction and Amplicon Sequencing

A modified magnetic beads protocol was used for extraction of stool DNA (TIANGEN Biotech, DP712). Following the manufacturer supplied protocol, 200 mg of the stool sample was transferred into a tube containing 500 μL of buffer solution SA (TIANGEN Biotech, DP712), 100 μL of buffer solution SC (TIANGEN Biotech, DP712), and 0.2 g of zirconia beads (NIKKATO, YTZ-0.2). The sample mixture was homogenized *via* the TGrinder H24 tissue-grinding homogenizer (TIANGEN Biotech) at a speed of 6 m/s for five sessions in 30-s intervals. DNA sample control was conducted based on having a DNA volume ≥200 ng, OD_260/280_ = 1.8–2.1, and a main band observed based on gel electrophoresis at >2000 bp.

The V4–V5 segments were amplified using the 515f-y/926r primer pair *via* PCR. The applied PCR settings were 98°C for 3 min, followed by 27 cycles at 98°C for 20 s, 55°C for 30 s, and 72°C for 30 s, and a final extension at 72°C for 2 min. PCR products were purified using AMPure XP Beads (Beckman, A63880) and amplified with Illumina P7 and P5 primer *via* PCR. The applied PCR settings were 98°C for 30 s, followed by 6 cycles at 98°C for 20 s, 60°C for 30 s, and 72°C for 30 s, and a final extension at 72°C for 2 min. The PCR products were again purified *via* AMPure XP Beads (Beckman, A63889). Library DNA was mixed with fluorescent dye (Qubit dsDNA HS Reagent) for quantitative quality control (with concentrations ≥2 ng/μL) using a Qubit 3.0 fluorometer. The DNA library fragment size was determined using a 2% agarose gel through electrophoresis and the library was determined to have no primer dimer contamination below 100 bp. The main band of the DNA library sample was determined to be 500 bp in size. The DNA library was subsequently pooled and sequenced *via* the Illumina MiSeq sequencing platform while applying the PE 300 protocol with overlapping reads.

#### Bioinformatics Processing and Analysis of Amplicon Sequencing Data

Amplicon sequences were bioinformatically processed using bioBakery workflows based on the DADA2 pipeline in R (Callahan et al., 2016; McIver et al., 2017). Specifically, sequences were first demultiplexed and run through DADA2 with default parameters to denoise, filter, and trim the sequences. Reads were then subject to sequence error rate learning and base-by-base error correction. Chimeras were then removed following dereplication, sample inference, and merging of paired end reads through DADA2 to yield an OTU table. Sequence alignment and phylogenetic tree reconstruction was subsequently conducted using Clustal Omega (Sievers et al., 2011). Lastly, taxonomic assignment was performed using the SILVA rRNA database (Quast et al., 2013).

### Statistical Analysis

Statistical analyses were performed *via* R. Normality of each measured variable is determined *via* the Shapiro–Wilk test. For variables that are normally distributed, two-tailed *t*-tests and Pearson’s correlation were used to compare values between groups and assess for correlations between variables. For variables that are not normally distributed, non-parametric statistics were performed using the Wilcoxon rank-sum test and Spearman’s rank correlation. The χ^2^-test was used to compare the gender composition between groups.

Linear discriminant analysis effect size (LEfSe) was used to elucidate the differentially abundant gut microbiota between groups (Segata et al., 2011). The LEfSe analysis was conducted using the one-against-all strategy for multi-class analysis with α = 0.05 for the factorial Kruskal–Wallis test between groups and a threshold of 2.0 for the logarithmic LDA score for discriminative features. MaAsLin2 was used to explore the per-feature correlations between clinical indices and microbial relative abundances (Mallick et al., 2021). The Wilcoxon rank-sum test was used to compare means of bacterial relative abundance between ASD and control groups and results were considered significant at a significance threshold of 0.05. False discovery rate (FDR) was computed to account for false positives due to multiple testing.

## Results

A total of 39 children with ASD, their corresponding mothers, and a cohort of healthy controls were enrolled, including 32 males and 7 females within the ASD group and 31 males and 13 females within the HC group. The mean age of ASD group was 4.74 ± 1.12 years (range 3.05–7 years) while the healthy control group was 5.11 ± 0.95 (range 3.01–6.52) years. To assess for associations between ASD and maternal factors, we additionally included each ASD subjects’ mother in this study. The mean age of the mother when giving birth to the ASD group subjects were 29.3 ± 4.5 years old. An overview of age distributions for ASD and healthy control subjects is shown in Supplementary Figure 1. No statistical differences in age nor gender were identified between the ASD and HC groups (*P* > 0.05, Table 1).

**TABLE 1:** Demographic information of the study participants.

	ASD (*n* = 39*)	Healthy control (*n* = 44)	Mother control (*n* = 38)	ASD-HC *P*-value
**Age (years)**				
Age (mean ± SD)	4.74 ± 1.12	5.11 ± 0.95	34.12 ± 5.07	0.11
Age range	3.05–7	3.01–6.52	21–45.59	
Age at delivery (mean ± SD)			29.3 ± 4.5	
**Sex**				
Male (*n*)	32	31	0	0.33
Female (*n*)	7	13	38	

*Two individuals with ASD are twins, sharing the same mother.

### Differences in Serum Oxytocin Levels and GIS Index Between Children With Autism Spectrum Disorder Compared and Healthy Controls in Male Subjects

**FIGURE 1:**
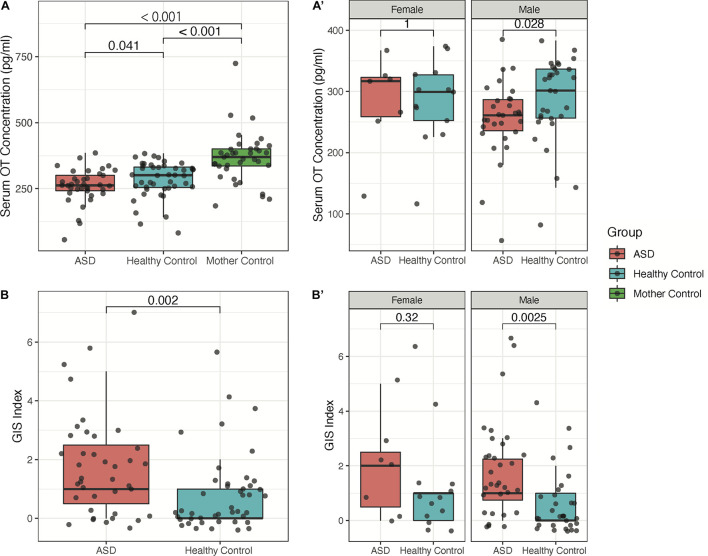
Sex-dependence of serum OT concentration and GIS index between children with ASD, their mothers, and healthy controls. **(A)** Groupwise comparison of serum OT concentration in all subjects. **(A′)** Groupwise comparison of serum OT concentration by sex. **(B)** Groupwise comparison of GIS index between children with ASD and healthy controls. **(B′)** Groupwise comparison of GIS index by sex.

The mean of serum OT levels in the 39 participants of the ASD group (260.53 ± 65.44 pg/ml) was significantly lower than that of the healthy control group (284.82 ± 68.37 pg/ml, *P* < 0.05, Figure 1). Moreover, the mothers’ serum OT levels were found to have a value of 367.42 ± 96.89 pg/ml and is found to be significantly higher in concentration than both ASD and HC groups (*P* < 0.001, Figure 1). Among males, we found that children with ASD have lower serum OT levels and more severe GIS index (*P* < 0.05, Figure 1); however, these effects are not observed in comparisons between groups among females, which is likely due to a small sample size of female children with ASD (*n* = 7).

### Different Gut Microbiota Compositions Between Children With Autism Spectrum Disorder and Healthy Controls

**FIGURE 2:**
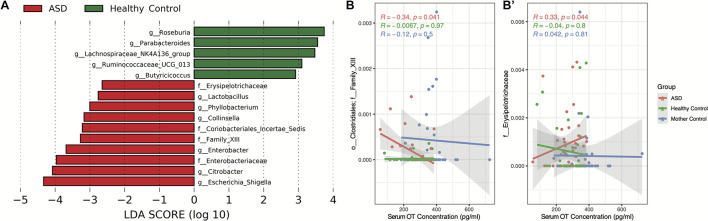
Differentially abundant microbiota between ASD and HC group individuals and associated correlations with serum oxytocin concentration. **(A)** LDA effect size (LEfSe) analysis was conducted to assess for differentially abundant taxa using the one-against-all strategy for multi-class analysis with α = 0.05 for the factorial Kruskal–Wallis test between groups and a threshold of 2.0 for the logarithmic LDA score for discriminative features. **(B,B′)** Spearman’s rank correlation of serum oxytocin concentration against significant differentially abundant taxa. Significant correlations using α = 0.05 are shown.

Differentially abundant gut microbiota between individuals with ASD and HC were assessed and visualized *via* LDA effect size (LEfSe). Significant differentially abundant microbiota between groups is shown in Figure 2A. In particular, we observed that the genera *Roseburia*, *Parabacteroides*, Lachnospiraceae NK4A136 group, Ruminococcaceae UCG-013, and *Butyricicoccus* are more abundant within the HC group (*P* < 0.05, Figure 2A). Similarly, the genera *Lactobacillus*, *Phyllobacterium*, *Collinsella*, *Enterobacter*, *Citrobacter*, and *Escherichia/Shigella* along with unidentified genera from the families Erysipelotrichaceae, Coriobacteriales Incertae Sedis, Clostridiales Family XIII, and Enterobacteriaceae are more abundant within the ASD group (*P* < 0.05, Figure 2A). Following the determination of differentially abundant genera between ASD and HC groups, we then assessed whether such gut microbes are correlated with serum OT concentration. We applied *MaAsLin2* to identify all correlations between gut microbiota abundance and serum OT levels in children with ASD and between the gut microbiota abundance of children with ASD and serum OT levels of mother controls, which did not yield any significant correlations following FDR-adjustment of *P*-values in either analysis. Furthermore, we performed correlational analysis using only the differentially abundant genera between ASD and HC groups *via* Spearman’s rank correlation without FDR-adjustment and found that the unidentified Clostridiales Family XIII (*R* = −0.34, *P* < 0.05) and unidentified Erysipelotrichaceae (*R* = 0.33, *P* < 0.05) genera are determined to be significantly correlated with serum OT concentration in children with ASD (Figures 2B,B′).

## Discussion

### Reduced Serum Oxytocin in Children With Autism Spectrum Disorder Compared to Healthy Controls

This study found that the serum level of OT in children with ASD was lower than that of healthy controls. This result is consistent with many previous studies of reduced OT levels in children with ASD of the similar ages and young adults with high functioning ASD (Andari et al., 2010; Husarova et al., 2015; Zheng et al., 2017; Aita et al., 2019). However, relevant studies in literature have implicated largely heterogenous findings within the present study age range. Specifically, Miller et al. reported no statistical differences in serum OT levels between 8 and 18 year old high-functioning children with ASD and controls (Miller et al., 2013), while Yang et al. (2017) reported elevated serum OT levels among children with ASD aged 2–17 years old. Such inconsistent results could be influenced by multiple factors, including the wide diversity of the ASD population with different genotypes and epigenetic factors as well as different methodologies of measurements. The reduction of OT levels in ASD is likely a result of both genetic and epigenetic changes related to its pathogenesis. For instance, past genetic studies have found that mothers and siblings of children with ASD had oxytocin receptor (OTR) gene deletions, while children with ASD did not have this gene deletion but displayed increased OTR methylation (Gregory et al., 2009). Furthermore, hypermethylation of OTR is associated with reduced OT levels (Dadds et al., 2014), which led to the associated reduction in gene expression and resulted in low production of OT. Several other mechanisms have also been postulated to cause a reduction in OT release, such as reduced OT levels due to CD38 mutation (Jin et al., 2007), abnormalities in OT prohormone processing (Green et al., 2001), as well as OTR abnormalities and reduction in the stimulation of endogenous secretion in response to positive events such as parental touch or negative events such as anxiety (Feldman et al., 2012; Taurines et al., 2014; Watanabe et al., 2017). Taken together, OT levels are not only related to genetic polymorphisms and mutations of OTR (Freeman et al., 2018), but are also influenced by multiple epigenetic factors impacting its expression, secretion, and processing. Since dysfunction of OT signaling was found to be implicated to impact the etiology of ASD, there have been many clinical trials aimed to administer OT in hopes of targeting the ASD core symptoms (Alaerts et al., 2020; Bernaerts et al., 2020; Procyshyn et al., 2020). However, the outcomes have been shown to be inconsistent and is thus not expected. Pretreatment OT level is an important influencing factor. Parker et al. (2017) reported that individuals with the lowest pretreatment OT concentrations show the greatest SRS total raw score improvement with intranasal OT treatment. The poor treatment response of those with elevated OT level is likely related to OT prohormone processing and receptor dysfunction with certain OT receptor polymorphisms, especially that of the OTR rs2254298, which is more commonly seen in Asian individuals (Watanabe et al., 2017; Yang et al., 2017; Freeman et al., 2018). Thus, future studies using more advanced OT functional measurements along with measurements of OTR could provide more significant clinical applications and warrant further studies in the field.

### Altered Gut Microbiota Composition Between Children With Autism Spectrum Disorder and Healthy Controls

To further our observations on the groupwise differences in serum OT concentration, we assessed the influence of gut microbiota on serum OT. Based on our findings, we hypothesize that significant negative correlation between OT level and gut microbiome dysbiosis which has not yet previously reported. Past studies have shown that the effects of gut microbiota may exert regulatory effects on host OT expression *via* the immune–endocrine-brain signaling network (Erdman and Poutahidis, 2016). Furthermore, OT also possesses potent anti-inflammatory effects through the reduction of interleukins (Clodi et al., 2008). Thus, we hypothesize that gut microbiota secretions and metabolites may influence host brain OT signaling bidirectionally, thereby suggesting an alternative pathway of mediating inflammatory responses in individuals with ASD. Based on findings of the present study, the HC group gut microbiome is characterized by several butyrogenic gut microbiota, including *Roseburia*, Lachnospiraceae NK4A136 group, Ruminococcaceae UCG-013, and *Butyricicoccus*. Previously, we have determined that the presence of gut butyrogenic microbiota are indications of good GI health and the abundances of such microbiota are associated with anti-inflammatory effects (Cao et al., 2021). Based on such findings, the detection of high abundances of butyrogenic bacteria within the HC group is thus consistent with expectations. In addition, *Parabacteroides* was also found to be significantly more abundant within the HC group, which is consistent with existing findings within literature (Xu et al., 2019). In contrast, the ASD group gut microbiome is characterized by an overgrowth of several pathogenic Enterobacteriaceae genera, including *Enterobacter*, *Citrobacter*, and *Escherichia/Shigella*. Interestingly, we found that the ASD group displays significantly higher abundances of an unidentified Erysipelotrichaceae genus, which was found to be positively correlated with serum OT levels. Provided that the Erysipelotrichaceae family consists of largely butyrogenic bacteria, this unidentified genus then represents the only known differentially abundant butyrogenic bacteria within the ASD gut microbiome. Interestingly, we also found that the heightened abundance of unidentified Clostridiales Family XIII genus within the ASD group is negatively correlated with serum OT concentration, which is suggestive of opposing effects on OT relative to that of the unidentified Erysipelotrichaceae genus. Thus, this new finding of that lower OT level correlated with higher levels of pathogenic microbiota and lower levels of butyrogenic microbiota in ASD group relative to HCs, which is indicative of a low OT level corresponding to gut microbiome dysbiosis. We have previously observed that ASD patients displaying similar gut microbiome profiles with heightened levels of proinflammatory cytokines to the extent of a cytokine storm (Cao et al., 2021). Furthermore, OT along and/or combination therapy with probiotics trial for ASD individuals achieved therapeutic effects in improving their social behaviors and gut dysbiosis (Kong et al., 2021). Taken together with the present results of reduced serum OT concentration and its correlation with gut microbiome dysbiosis, such results supported the rationale for OT treatment in ASD patients; the proposed bidirectional regulation of dysbiotic gut microbiota on host brain OT signaling can indirectly modulate immune cytokine activation among individuals with ASD. Of note, the level of microbiome dysbiosis and ASD severity based on ADOS scores were not checked in this study, which could be further studied. We believe that measurements of OT levels play a significant role in elucidating the potential subtypes of ASD and may predict treatment response, although a more accurate measurement including OT processing level is warranted in future studies. With our further understanding of OT signaling mechanisms and their relationship with blood OT concentrations, we consider these factors as important influencing factors in the design of further studies to identify how to more effectively improve OT signaling in different ASD subsets and phenotypes.

### Potential Sex-Related Differences in Oxytocin Expression Between Children With Autism Spectrum Disorder and Healthy Controls

Based on the results of the present study, we additionally hypothesize that sex-dependent mechanisms of OT expression may contribute to the differences in severity of GI symptoms between children with ASD and healthy controls. In this study, we found that the observed trend of lower OT level in children with ASD relative to HC was only significant among male individuals with ASD but not among female individuals. Notably, we would like to acknowledge the small female sample size in this study, which may not provide sufficient statistical power for the generalization of interpreted results; however, we believe that such findings may serve as preliminary evidence for the potential sex-dependent differences in OT expression between children with ASD and healthy controls. Specifically, we observed greater GI symptom severity among males with ASD when compared to males in the healthy control group with no differences observed between groups among females, which have not been previously reported. Such sex-dependent differences in serum OT concentration are consistent with other previous studies, as several recent studies using animal models and in humans have found that females find same-sex social interactions more rewarding than males (Feng et al., 2015; Borland et al., 2019). Therefore, it is likely that such rewarding attributes from social interaction arise from higher expression levels of OT at baseline among females. Interestingly, these differences coincide with the observed differences in GIS index, as we found that males with ASD also have more severe GI symptoms as compared to male healthy controls. As previously discussed, OT is a potent anti-inflammatory molecule and may have the potential to alleviate several GI conditions with inflammatory causes that are commonly associated with ASD. In particular, inflammatory bowel disease (IBD) has been previously determined to be associated with ASD and was demonstrated to have associations with the behavioral severity of ASD (Welch et al., 2005; Lee et al., 2017). Thus, it is likely that differences in OT expression mediates both the inflammatory causes of GI disorders observed in children with ASD as well as associated psychosocial and behavioral symptoms of ASD. However, the current study provides limited direct evidence on such associations as no significant correlations between serum OT levels and GI symptom severity were observed (data not shown).

There are several limitations of the study worth considering based on the present study. First, the relatively small sample size limited our ability to perform further subgroup analyses based on ASD core symptom severity. Second, the subject sample consisted of a homogenously Chinese group of individuals, such that the present results may not be generalizable to the broader ASD population. Lastly, females are likely underrepresented in our dataset, as only 7 female children with ASD were enrolled relative to the 32 male children with ASD; however, we believe that the male-to-female ratio of enrolled subjects in the present study (4.6:1) is representative of the sex ratio within ASD population, which has previously been reported to be 4.2:1 (Fombonne, 2009). Nonetheless, further studies with a balanced sex distribution may provide greater evidence in the elucidation of sex-dependence in OT expression.

Our findings in the present study corroborate a potential role of peripheral blood OT levels and associated changes in the gut microbiota composition in children with ASD. Lower serum OT levels were found in children with ASD when compared to that of healthy controls and potential sex-related differences in serum OT levels and GIS index were observed. Additionally, children with ASD also showed trends of higher levels of gut microbiome dysbiosis associated with lower OT levels. The findings of the present study provide suggestive evidence for our understanding of the clinical significance of OT blood level and its role and relation with OT signaling in ASD, which warrants further large-scale studies in this direction to further validate the present findings and explore the predictive factors for the therapeutic response of OT in the subset of ASD individuals.

## Data Availability Statement

The datasets presented in this study can be found in online repositories. The names of the repository/repositories and accession number(s) can be found below: https://www.ncbi.nlm.nih.gov/bioproject/687773, PRJNA687773.

## Ethics Statement

The studies involving human participants were reviewed and approved by Shenzhen Maternity and Child Healthcare Hospital. Written informed consent to participate in this study was provided by the participants’ legal guardian/next of kin.

## Author Contributions

X-JK and GW conceived the concept, developed the experimental design, and provided the resources and major funding. MH, ZW, ZF, JC, JY, and QZ contributed to data collection. KL, MH, and X-JK finished the first draft and major revisions of the manuscript. KL contributed on data analysis and creation of figures and tables. All authors have read and agreed to the published version of the manuscript.

## Conflict of Interest

The authors declare that the research was conducted in the absence of any commercial or financial relationships that could be construed as a potential conflict of interest.

## Publisher’s Note

All claims expressed in this article are solely those of the authors and do not necessarily represent those of their affiliated organizations, or those of the publisher, the editors and the reviewers. Any product that may be evaluated in this article, or claim that may be made by its manufacturer, is not guaranteed or endorsed by the publisher.

## Footnotes

1
https://www.biomart.cn/infosupply/59585234.htm
