# Supplementary material for: Serum Oxytocin Level Correlates With Gut Microbiome Dysbiosis in Children With Autism Spectrum Disorder
Source: Front Neurosci. 2021 Oct 1;15:721884. doi: 10.3389/fnins.2021.721884 (PMC8517432; doi:10.3389/fnins.2021.721884)
Supplement: Supplementary file 1 [file Image_1.pdf]

Supplementary Material

Supplementary Figures

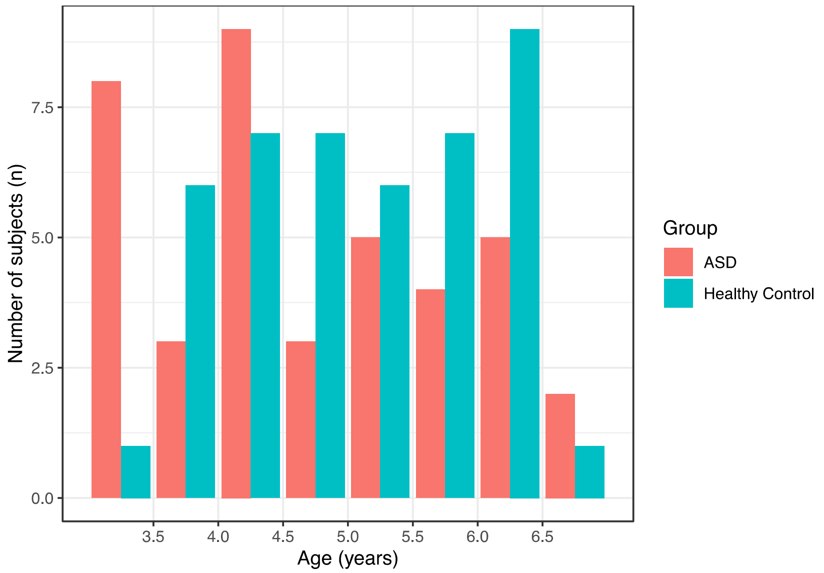

Supplementary Figure 1. Overview of study participant age distribution for ASD and healthy control group subjects.
